# Advances in Virus Detection Techniques Based on Recombinant Polymerase Amplification

**DOI:** 10.3390/molecules29204972

**Published:** 2024-10-21

**Authors:** Shiwen Wu, Wenhan Yu, Xianshu Fu, Xiaoping Yu, Zihong Ye, Mingzhou Zhang, Yulou Qiu, Biao Ma

**Affiliations:** Key Laboratory of Microbiological Metrology, Measurement & Bio-product Quality Security, State Administration for Market Regulation, College of Life Sciences, China Jiliang University, Hangzhou 310018, China; s22090710056@cjlu.edu.cn (S.W.); s23090710071@cjlu.edu.cn (W.Y.); yxp@cjlu.edu.cn (X.Y.); zhye@cjlu.edu.cn (Z.Y.); zmzcjlu@cjlu.edu.cn (M.Z.); yulou@cjlu.edu.cn (Y.Q.); mb@cjlu.edu.cn (B.M.)

**Keywords:** isothermal amplification, recombinant polymerase amplification (RPA), multi-technique combination, virus detection

## Abstract

Recombinase polymerase amplification (RPA) has emerged as a rapid, efficient, and highly sensitive method for nucleic acid amplification, thus becoming a focal point of research in the field of virus detection. This paper provides an overview of RPA, emphasizing its unique double-stranded DNA synthesis mechanism, rapid amplification efficiency, and capability to operate at room temperature, among other advantages. In addition, strategies and case studies of RPA in combination with other technologies are detailed to explore the advantages and potential of these integrated approaches for virus detection. Finally, the development prospect of RPA technology is prospected.

## 1. Introduction

In recent years, several viruses have emerged and spread epidemically, including the avian influenza virus (AIV) [1] in 2013, the Zika virus (ZIKV) [2] in 2015, the Ebola virus (EBOV) [3] in 2018, the African swine fever virus (ASFV) [4], COVID-19 [5] in 2019, and the influenza A virus (IAV) [6] in 2023. Although biosecurity measures and the development of new vaccines have significantly contributed to the prevention of these viral infections, they still pose serious threats to human health and animal and food safety, resulting in substantial losses in the livestock industry. Isothermal amplification technology has gained significant attention due to its rapidity, simplicity, and elimination of complex temperature cycling and instrumentation requirements [7]. Notable techniques in this category include recombinase polymerase amplification (RPA) [8], recombinase-aided amplification (RAA) [9], loop-mediated isothermal amplification (LAMP) [10], nucleic acid sequence-based amplification (NASBA) [11], strand displacement amplification (SDA) [12], rolling circle amplification (RCA) [13], and helicase-dependent amplification (HDA) [14]. Among these techniques, RPA is highly valued due to its ease of use, rapid speed, and high sensitivity, enabling quick detection in the field [15]. Recent studies have shown that combining RPA with other technologies enhances its diagnostic performance, resulting in faster, more sensitive, and more accurate outcomes compared to traditional RPA alone. These techniques include fluorescence signal detection [16], lateral flow dipsticks (LFD) [17], clustered regularly interspaced short palindromic repeats (CRISPR)/CRISPR-associated (Cas) systems [18], flocculation assays detection [19], electrochemical detection [20], chemiluminescent detection [21], surface-enhanced Raman scattering (SERS) [22], surface-enhanced infrared absorption spectroscopy (SEIRA) [23], and microfluidic technology [24].

## 2. Traditional Virus Detection Techniques

Traditional virus detection techniques include virus isolation and identification, polymerase chain reaction (PCR), and enzyme-linked immunosorbent assay (ELISA). However, these techniques often present several drawbacks, including cumbersome procedures, lengthy detection times, and relatively low sensitivity [25].

### 2.1. Virus Isolation and Identification

Virus isolation and identification involve processing blood, body fluids, feces, tissues, and organs from infected animals, followed by the expansion and culture of the virus using methods such as cell culture, animal inoculation, and chicken embryo inoculation [26]. This method is often regarded as the “gold standard” for most virus detection and has the advantage of accurate detection [27,28]. However, virus isolation and culture typically require a significant amount of time, must be performed in a sterile environment [29], and involve complex procedures. Moreover, the stringent requirements for both personnel and laboratory conditions render this approach unsuitable for rapid pathogen detection and impractical for detecting large volumes of samples [30].

### 2.2. Real-Time Fluorescence Quantitative PCR (qPCR)

In 1992, Higuchi et al. [31] combined PCR technology with closed detection methods to quantitatively analyze the amount of target nucleic acid, thus introducing the concept of fluorescence quantitative PCR technology. In 1995, the PE Company in the United States successfully developed TaqMan technology, and in 1996, Applied Biosystems introduced the first fluorescence quantitative PCR detection system [32]. Real-time fluorescent quantitative PCR involves the addition of fluorescent substances to the PCR reaction system, allowing for the monitoring of the entire PCR process in real-time through fluorescence signals. This enables the quantitative analysis of unknown starting templates [33]. However, real-time fluorescent quantitative PCR necessitates specialized thermal cyclers and reagents, which can be quite costly. Factors such as the presence of homologous and heterologous DNA backgrounds [34], the specificity of oligonucleotide hybridization [34], the ratio of TaqMan probes [34], the concentration of SYBR Green I [35], and the length of PCR products [35] can all contribute to quantitative deviations in real-time fluorescence quantitative PCR reactions.

### 2.3. Enzyme-Linked Immunosorbent Assay (ELISA)

ELISA was introduced in 1971 [36]. In this method, antigens and antibodies are adsorbed onto the surface of solid-phase carriers, followed by labeling these antigens and antibodies with enzymes [37]. The results are then determined through the reaction of the antigens or antibodies on the solid-phase carriers [38]. Despite its widespread use, ELISA has several disadvantages, including high costs, lengthy detection times [39], a high probability of cross-reactivity [40], and its unsuitability for rapid on-site detection [41].

## 3. Isothermal Amplification Techniques

Since the early 1990s, various isothermal nucleic acid amplification techniques have emerged, with recombinant polymerase amplification (RPA) technology being one of the fastest-developing techniques in this field. Despite its relatively late introduction, RPA has experienced rapid adoption and commercialization.

### 3.1. Recombinase Polymerase Amplification (RPA)

**Figure 1:**
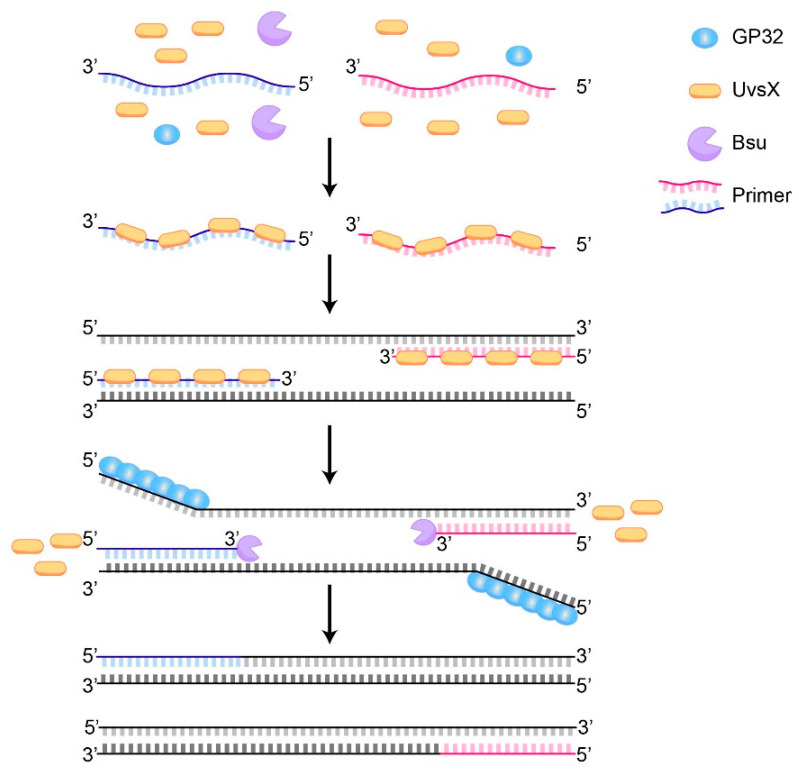
Schematic diagram of recombinant polymerase amplification.

Recombinase polymerase amplification (RPA) is an isothermal amplification technology introduced by the British company Twist DX in 2006 [42]. The principle of RPA is illustrated in Figure 1. As depicted, the reaction system primarily consists of several key enzymes, including the recombinase (T4 UvsX), single-stranded DNA-binding protein (T4 Gp32), and strand-displacing DNA polymerase (Bsu) [43]. During the reaction, the recombinase binds to the oligonucleotide primer, forming a complex of the enzyme and primer [44]. This complex facilitates the primer’s localization to the homologous target sequence of the double-stranded DNA template. The single-stranded DNA-binding protein (T4 Gp32) aids in unstranding the template DNA [42]. Subsequently, the recombinase dissociates from the primer, allowing the Bsu polymerase to bind to the 3′ end of the primer and initiate the synthesis of a new DNA strand [45]. This process is repeated, achieving the exponential amplification of the target DNA sequence.

The reaction process of RPA does not require significant temperature changes and can be conducted at temperatures ranging from 37 to 42 °C. This feature offers clear advantages over other isothermal amplification techniques [46]. Notably, the RPA primer design does not have specific annealing temperature requirements [47]. Additionally, the reaction time is short, allowing for detection within 5 to 20 min [48]. The operation is straightforward, and RPA does not require specialized instruments, making it suitable for instant detection [49]. Furthermore, RPA exhibits some tolerance to mismatches and inhibitors [44]. Multiple detections can be performed for different pathogens or genes within a single assay [50], and RPA can be effectively combined with various detection technologies [51]. Overall, RPA is a promising nucleic acid amplification technology [52].

RPA has clearly demonstrated its advantages and potential in clinical diagnostics. Ying et al. [53] proposed a novel recombinase polymerase amplification detection method (RT-RPA), which is designed for the typing detection of two subtypes of human papillomavirus (HPV), 16 and 18. RPA is also widely utilized in the field of food safety. Li et al. [54] employed RPA technology for the isothermal amplification of the invA gene of *Salmonella*, achieving direct quantitative measurement through photocolorimetry. RPA technology is also well-suited for detecting pathogens in agriculture and livestock. Chen et al. [55] optimized the RT-RPA detection method for the identification of wheat yellow mosaic virus and Chinese wheat mosaic virus. Aebischer et al. [56] developed an RPA method for the simultaneous detection of bovine viral diarrhea virus and Schmallenberg virus. Additionally, Amer et al. [57] established an RT-RPA method for the detection of bovine coronavirus. The application of RPA technology to detect viruses is shown in Table 1.

However, RPA also has its limitations and challenges. In the process of nucleic acid extraction and sample addition, repeated sample addition or open-cap shock reaction tubes are prone to produce aerosol contamination [69]. To address this issue, Arizti-Sanz et al. [70] integrated the RPA and Cas13 systems into a single reaction tube, allowing reactions to proceed without opening the tube, thereby minimizing aerosol contamination. Currently, there is no specialized software available for designing RPA primers, making the primer and probe design process a significant challenge. To tackle this problem, Higgins et al. [71] proposed the development of primer design software (PrimedRPA), which automatically selects RPA primers and probes. This software compares multiple pairs of primers and probes to identify conserved sequences while filtering out regions that may cross-react with background organisms, thus addressing the issue of mismatching between primers and probes. Since RPA amplifies nucleic acids at a single temperature, it cannot avoid binding between primers through heating cycles, which can lead to the amplification of non-target bands. This is particularly problematic in scenarios with no-template or low-template concentrations, as it may reduce reaction efficiency and negatively impact experimental outcomes. To mitigate this issue, Sharma et al. [72] developed a self-avoiding molecular recognition system (SAMRS) designed to prevent the formation of primer dimers. Despite these challenges, the unique advantages of RPA make it highly suitable for field testing in resource-limited environments, and it is expected to become a powerful tool for on-site diagnostics.

### 3.2. Other Isothermal Amplification Techniques

Other isothermal amplification technology include RAA, LAMP, NASBA, SDA, RCA, HDA. And the differences between RPA and other isothermal amplification techniques are shown in Table 2.

#### 3.2.1. Recombinase-Aided Amplification (RAA)

Recombinase-aided amplification (RAA) is a nucleic acid isothermal amplification technology developed by Chinese scholars in 2010 [73]. This technique primarily relies on the action of three proteins: a recombinase, a single-stranded binding protein, and DNA polymerase [74]. During the RAA process, the recombinase and the single-stranded binding protein bind to the primer in the presence of ATP, forming a recombinase-single-stranded binding protein–primer complex. This complex then scans the template DNA strand; when a complementary sequence is identified, a stable D-loop is formed. Under conditions of sufficient deoxyribonucleoside triphosphates (dNTPs) and ATP, DNA completes the extension of the DNA chain at constant temperature and forms a new DNA chain [75]. The entire amplification process typically occurs at 37 °C for 15 to 30 min [73], yielding a significant number of amplified products that increase exponentially. However, designing primers remains a challenge, as there is currently no dedicated software for RAA primer and probe design, which limits the widespread application of this technology [76].

#### 3.2.2. Loop-Mediated Isothermal Amplification (LAMP)

Among isothermal amplification techniques, loop-mediated isothermal amplification (LAMP) is one of the most extensively studied methods, originally designed by Notomi et al. [77]. Four special primers were designed for six regions of the target gene [78], employing Bst DNA polymerase with chain-displacing activity for efficient amplification at a constant temperature of 60 to 65 °C [78]. The LAMP process includes three stages: template synthesis, cyclic amplification, and prolonged recirculation [79]. It is characterized by its high sensitivity, strong specificity, and ease of product detection [80]. However, LAMP requires stringent primer design [81], and false positives can occur during operation [82].

#### 3.2.3. Nucleic Acid Sequence-Based Amplification (NASBA)

Nucleic acid sequence-based amplification (NASBA) was first reported by Compton in 1991 [83]. This technique operates using three enzymes: AMV reverse transcriptase, ribonucnase H (RNase H), and T7 RNA polymerase [84]. NASBA employs RNA as a template for reaction with two specific primers, achieving sensitivity down to a single copy of the target molecule [85,86]. Although NASBA is primarily designed for detecting RNA sequences, it can also be adapted for DNA detection by introducing two denaturation steps [87]. However, the complexity of its reaction composition and the requirement for three enzymes contribute to higher costs [82].

#### 3.2.4. Strand Displacement Amplification (SDA)

Strand displacement amplification (SDA) was first proposed by Walker et al. in 1992 as a method for isothermal DNA amplification through enzymatic reactions [88]. The SDA process consists of three stages: preparation of a single-stranded DNA template, generation of a target DNA fragment, and a strand displacement reaction [89]. Under isothermal conditions, the method can amplify the target sequence by factors of 10^9^ to 10^10^ in a relatively short amount of time [90]. However, SDA requires costly enzymes and non-standard nucleotides, and endonuclease recognition sequences are present at both ends of the amplified products [91].

#### 3.2.5. Rolling Circle Amplification (RCA)

Rolling circle amplification (RCA) was first utilized in the mid-1990s to synthesize copies of circular nucleic acid molecules, such as plasmids [92]. This technique employs a circular template along with phi29 DNA polymerase to convert dNTPs into single-stranded DNA using a short DNA primer [93]. RCA can directly amplify both DNA and RNA [94], and it can also facilitate signal amplification of the target nucleic acid, achieving sensitivity down to a single copy of the nucleic acid molecule [95]. While RCA requires nucleic acid extraction, its steps are complex, involving extensive sample pretreatment and a longer detection time, which limits its application range [96].

#### 3.2.6. Helicase-Dependent Amplification (HDA)

Helicase-dependent amplification (HDA) is an isothermal amplification technology introduced by the New England Biolabs (NEB) in 2004 [97]. This process involves the dissociation of the DNA double-strand by helicase at a constant temperature, followed by the stabilization of the single-stranded DNA by single-stranded binding protein, which provides a template for primers [98]. Subsequently, DNA polymerase synthesizes complementary double strands [99], and this cycle is repeated continuously, resulting in exponential amplification of the target sequence. The amplification reaction typically takes 75 to 90 min [100] and is conducted at a constant temperature of 60 to 65 °C [101]. However, the reaction system of this method is relatively complex, and method optimization is constrained by this system. Most of the reaction process needs to be completed in two steps at two different temperature conditions, with reaction times typically ranging from 1 to 2 h, which may limit its usefulness for certain applications [102].

**Table 2:** Differences between RPA and other isothermal amplification techniques.

Reaction	Reactive Enzymes	LOD	Primers	Reaction Temperature	Reaction Time	Template	Ref
RPA	recombinase (T4 UvsX), single-stranded DNA-binding protein (T4 Gp32), strand-displacing DNA polymerase (Bsu)	10 copies/μL	1 pair	37~42 °C	5~20 min	DNA	[15]
RAA	recombinase, single-stranded binding protein, DNA polymerase	High	1 pair	37 °C	15~30 min	DNA	[103]
LAMP	Bst DNA polymerase	10 copies/μL	2~3 pairs	60~65 °C	30~60 min	DNA	[104]
NASBA	AMV reverse transcriptase, ribonucnase H (RNase H), T7 RNA polymerase	100 CFU/mL	1 pair	40~55 °C	90~120 min	DNA/RNA	[105]
SDA	restriction endonuclease enzymes, DNA polymerase	High	2 pairs	40~55 °C	15~20 min	DNA	[106]
RCA	phi29 DNA polymerase	High	1 primer or 1 pair	37 °C	60 min	DNA/RNA	[93,95]
HDA	helicase, single-stranded binding protein, DNA polymerase	High	1 pair	60~65 °C	75~90 min	DNA	[107]

## 4. Combined Application of RPA with Other Techniques

### 4.1. Real-Time Fluorescent RPA

Real-time fluorescence RPA integrates exonuclease III (exo) and an exonuclease fluorescent probe into the basic RPA reaction system, enabling real-time monitoring of template amplification [108]. The exo-fluorescent probe is composed of a fluorescence reporter group, a fluorescence quenching group, and an abasic site (typically a tetrahydrofuran site) [109]. When the probe binds to the amplified target DNA, exonuclease III cleaves at the cleavage site, separating the fluorophore from the quenching group. This reaction generates a fluorescence signal that is proportional to the amount of amplified target DNA, with fluorescence intensity increasing as amplification progresses [110]. Yang et al. [111] developed a novel fluorescent probe based on RPA for detecting Orf virus (ORFV). Their assay demonstrated the capability to detect as low as 10^2^ copies of ORFV DNA per reaction and exhibited high specificity, showing no cross-reaction with closely related viruses. Furthermore, the results correlated well with quantitative PCR (qPCR) outcomes. Wang et al. [112] combined RPA with real-time fluorescence detection to establish a method for detecting porcine parvovirus (PPV), achieving results within 20 min. This method had a detection limit of 10^3^ copies, found to be 100% consistent with results from real-time fluorescence quantitative PCR. One significant advantage of the real-time fluorescent RPA process is that it eliminates the need to open the reaction lid, thereby reducing the risk of aerosol contamination and false positives [113]. Additionally, fluorophore labeling on the primer helps prevent false-positive signals caused by primer dimers [114], significantly improving detection accuracy.

### 4.2. RPA-LFD

**Figure 2:**
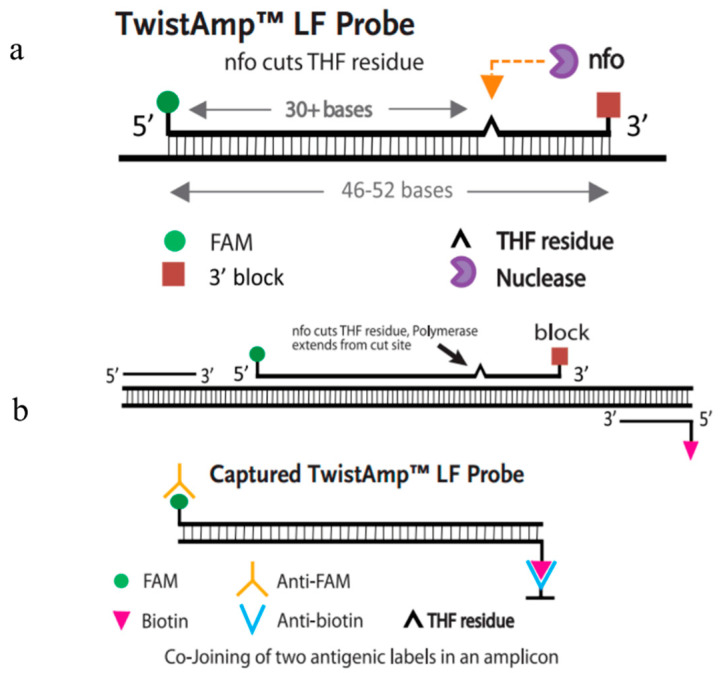
Schematic diagram of the Twist Amp^®^ RPA-LFD reaction (Quoted from Twist Amp^®^ DNA Amplification Kits, Scarborough, NY, USA). (**a**) Schematic diagram of the Twist Amp^®^ RPA-LFD probe. (**b**) Schematic representation of the reaction between the Twist Amp^®^ RPA-LFD probe and primer.

RPA-LFD is an innovative technique that combines the amplification principle of RPA with the detection capabilities of lateral flow assays. This method incorporates exonuclease IV (nfo) and a nfo probe into the basic RPA reaction system [115]. The Twist Amp nfo kit, developed by Twist DX in the UK, is widely utilized in this research area [116]. The detection principle, illustrated in Figure 2, involves several key components: the 5′ end of the downstream primer is labeled with biotin, the 5′ end of the probe is labeled with carboxyfluorescein (FAM), and the 3′ end of the probe is modified with a blocking group (C3-spacer) [109]. During the RPA-LFD reaction, target nucleotides are specifically amplified using the FAM-labeled probes and biotin-labeled primers, resulting in the production of amplification products that carry both labels (FAM and biotin). These amplification products subsequently diffuse and form a ternary complex with colloidal gold-labeled anti-FAM antibodies. This complex then binds to the detection line of the LFD, which contains biotin antibodies, generating a visible red signal. In contrast, probes that are not amplified by the primers will form a binary complex (lacking biotin) with the colloidal gold-labeled anti-FAM antibodies, which cannot bind to the biotin antibodies on the detection line [117].

Several studies have utilized RPA-LFD for specific pathogen detection. For instance, Hou et al. [118] designed RPA primers and probes targeting the conserved UL52 region of the infectious bovine rhinotracheitis virus (IBRV). This method enables detection at 38 °C within 25 min, achieving a detection limit of five copies per reaction and no cross-reactivity with other viruses causing gastrointestinal or respiratory infections in cattle. Wu et al. [119] established an RPA-LFD approach for the detection of Epizootic hemorrhagic disease virus (EHDV) and the Palyam serogroup viruses (PALV), achieving analytical sensitivities of 7.1 and 6.8 copies/µL, respectively. Yang et al. [120] developed an RPA-LFD method for Orf virus (ORFV) detection, which can be completed within 25 min, demonstrating a detection limit of 80 copies per reaction and high specificity without cross-reactivity with the capripox virus, foot-and-mouth disease virus and peste des petits ruminants virus. Gao et al. [121] utilized RPA-LFD technology to create a highly sensitive and specific detection method for Porcine Deltacoronavirus (PDCoV), capable of completing detection within 10 min at 37 °C. This method exhibited a sensitivity that is 10 times greater than traditional PCR, with a detection limit of 10^2^ copies/µL. Miao et al. [122] detected the African swine fever virus (ASFV) using the RPA-LFD approach, attaining a sensitivity of 150 copies in under 10 min and showcasing high specificity toward ASFV.

### 4.3. RPA-CRISPR/Cas

CRISPR is a defense system that bacteria or archaea developed during evolution. It was named in 2002 by a team led by Jansen, who also discovered CRISPR-associated proteins (Cas) that are closely related to CRISPR [123]. Gasiunas et al. [124] demonstrated the capability of the CRISPR/Cas system to achieve gene editing in bacteria, viruses, and human cells, significantly increasing interest in this technology. In 2016, a research team led by Feng Zhang at the Massachusetts Institute of Technology identified that the Cas13 protein possesses the ability to cut RNA in a nonspecific manner [125], leading to the development of nucleic acid molecular diagnostic technologies based on the CRISPR/Cas13 system [126]. As a result, the application of the CRISPR/Cas system in molecular detection has expanded considerably. Currently, Cas9, Cas12a/12b, and Cas13a are the most commonly used proteins in conjunction with CRISPR [127]. Additionally, Cas13b and Cas14a can also facilitate nucleic acid detection, although their applications are still relatively limited. When using the CRISPR/Cas system in isolation, the detection results may be inaccurate due to off-target effects or misidentification of Cas proteins [128]. However, integrating the CRISPR/Cas system with amplification technologies can enhance the accuracy of target gene identification, improve sensitivity and specificity, and reduce the likelihood of off-target effects.

**Figure 3:**
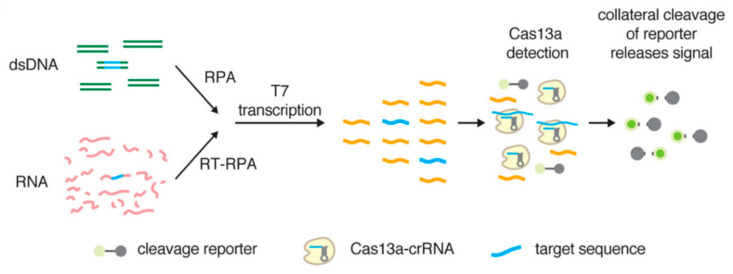
Schematic diagram of the SHERLOCK detection method [126].

In 2017, Professor Feng Zhang’s team developed a method known as specific high-sensitivity enzymatic reporter unlocking (SHERLOCK) for detection purposes. The detection principle, illustrated in Figure 3, involves the exponential amplification of target nucleic acids via RPA or reverse transcription RPA (RT-RPA), followed by reverse transcription into RNA. Guided by CRISPR RNA (crRNA), the Cas13a protein specifically identifies the target RNA, which activates the collateral cleavage activity of Cas13a. Di-nucleotide motifs labeled with fluorescence and quenching groups are cleaved, resulting in the emission of fluorescence from the reaction system. This emitted fluorescence signal is captured to enable the detection of target genes [127]. The SHERLOCK methodology successfully identified viral particles from Zika virus (ZIKV) and dengue virus (DENV) at concentrations as low as 2 attomolar (aM), allowing for differentiation between the two viruses [126]. Ren et al. [129] developed an RPA-CRISPR/Cas13a assay for detecting African swine fever virus (ASFV), demonstrating sensitivity down to a single copy and superior sensitivity compared to traditional qPCR, with no cross-reactivity to other swine viruses. Li et al. [130] employed RPA-CRISPR/Cas13a to detect the vp7 gene of grass carp reovirus (GCRV) type 1, achieving a detection limit of 7.2 × 10^1^ copies/μL. The detection process can be completed within one hour, and results are consistent with those of real-time fluorescence quantitative PCR, exhibiting no cross-reactivity with other common aquatic pathogens. Furthermore, the Doudna research team at the University of California demonstrated that Cas12a can be effectively combined with RPA for highly sensitive and specific diagnostic applications [131]. Ma et al. [132] successfully established a diagnostic platform for Senecavirus A (SVA) using RPA-CRISPR/Cas12a technology, achieving a minimum detection limit of 10 copies, with results showing 100% agreement with RT-qPCR data. Wang et al. [133] developed a highly specific detection method utilizing RPA-CRISPR/Cas12a for three viral pathogens: SARS-CoV-2, influenza A, and influenza B. This detection method can be completed within one hour, making it faster than many standard methods, and demonstrates a detection limit of approximately 10^2^ copies/μL, exhibiting no cross-reactivity with other common respiratory pathogens.

### 4.4. RPA Combined with Flocculation Assay Detection

**Figure 4:**
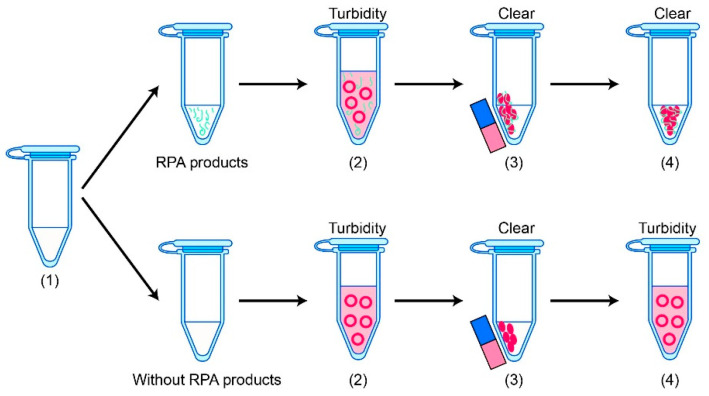
RPA in combination with flocculation assay detection. (1) RPA amplification; (2) RPA product capture by magnetic beads; (3) Magnetic beads adsorption; (4) Flocculation sedimentation. The RPA amplicons are incubated with magnetic beads in a low-pH buffer. Consequently, the precipitated RPA amplicons on the surfaces of the magnetic bead cross-link with multiple other RPA-magnetic bead conjugates, leading to flocculation and precipitating out of the solution, causing a sharp transition between solution phase and flocculate. In contrast, RPA reactions that lack a target template or contain non-target templates do not produce long DNA polymer segments.

Flocculation assay detection is based on a bridging flocculation phenomenon in colloid chemistry [134]. The fundamental principle of bridging flocculation involves utilizing long polymers to cross-link multiple particles, leading to their precipitation from a solution under specific buffering conditions [135], as illustrated in Figure 4. Healy and La Mer initially observed that long polymers could bind to multiple particles, triggering precipitation [136]. Later, in 1994, researchers discovered that carboxyl polymers could adsorb nucleic acids when utilized in a polyethylene glycol/sodium chloride (PEG/NaCl) solution [137]. In this context, magnetic beads can capture RPA products in a PEG/NaCl solution and gather under magnetic force. Wee et al. [138] were among the first to establish a method that combines flocculation detection with the RPA reaction, demonstrating its effectiveness. Flocculation is triggered by the presence of amplicons that are 100 nucleotides or longer. Specifically, RPA amplicons are incubated with magnetic beads in a low pH-buffered environment, resulting in a total detection time of just 10 min. When the amplified DNA fragment is equal to or greater than 100 bp, its binding to the magnetic beads facilitates cross-linking behavior, inducing flocculation. In contrast, short DNA primer pairs or limited amounts of DNA templates are insufficient for effective cross-linking with magnetic beads [139]. Hu et al. [19] combined the RPA reaction with polymer flocculation precipitation to develop a rapid, sensitive, specific, and user-friendly visual detection method for Staphylococcus aureus, achieving the capability to detect genomic DNA as low as 13 fg.

### 4.5. RPA Combined with Electrochemical Detection

By integrating RPA with an electroactive medium, this electrochemical method enables accurate detection of DNA in the field using a low-cost, portable electrochemical analyzer. Tsaloglou et al. [140] successfully employed [Ru(NH_3_)^6^]^3+^ as the electroactive medium for electrochemical detection of DNA, demonstrating the effective coupling of electrochemical detection and RPA within the same device. This method achieved the detection of genomic DNA at concentrations as low as 0.04 ng/μL in each sample. Chen et al. [141] labeled the upstream primers with electrochemiluminescence reagent (Ru-NHS ester) and the downstream primers with biotin to facilitate RPA amplification. The amplified products were then enriched using avidin-magnetic microspheres under a magnetic field. This system allowed for the detection of human papillomavirus 16 DNA through paper-based electrochemiluminescence detection technology, achieving a minimum detection limit of 0.05 copies/μL, which is 16 times more sensitive than real-time fluorescent PCR. Xia et al. [142] utilized the plasmid, including the hepatitis B virus (HBV) gene fragment, as a template, labeling the upstream primers with tripyridine ruthenium and the downstream primers with biotin to yield double-labeled amplicons via RPA. These amplicons were separated using streptavidin magnetic microspheres, and the tripyridine ruthenium signal was detected via paper-based electrochemiluminescence (ECL), achieving a detection limit of 1.2 pg/mL. Kim et al. [143] utilized reaction conditions for RPA near body temperature to develop a wearable detection device based on a multi-microelectrode array electrochemical biosensor. This device successfully detected the SARS-CoV-2 genome with limits of 0.972 fg/μL for the RdRP gene and 3.925 fg/μL for the N gene within 40 min. Despite these advancements, the main challenges associated with electrochemical technology include poor stability and limitations in field applicability. However, the use of microprocessing and nanoprocessing technologies presents an opportunity to miniaturize electrochemical biosensors, making them more suitable for field testing [144].

### 4.6. RPA Combined with Chemiluminescent Detection

**Figure 5:**
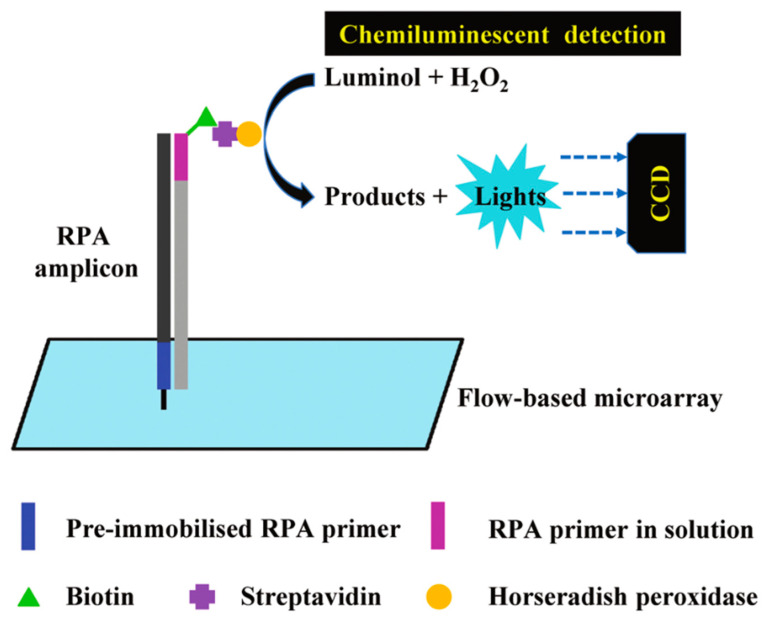
Schematic diagram of RPA combined with chemiluminescent detection [44].

The chemiluminescent detection converts chemical energy into the emission of visible light (luminescence) as the result of an oxidation or hydrolysis reaction [42]. For instance, the conversion of energy from oxidation between luminol and peroxide is catalyzed by horseradish peroxidase to give off luminescent signals detected by a charge-coupled device (CCD) camera. This principle is shown in Figure 5. Several researchers have successfully integrated RPA with chemiluminescent detection for applications in microbial detection using flow microarrays. Kunze et al. [145] employed RPA on a chip to facilitate the simultaneous amplification and detection of viral and bacterial DNA through flowing chemiluminescence microarrays. This method enabled the spatial isolation of DNA amplification responses for two water hygiene-associated viruses (Human adenovirus 41 and Phi X 174) and the bacterium Enterococcus faecalis, achieving detection limits of 35 GU/μL, 1 GU/μL, and 5 × 10^3^ GU/μL (genomic units), respectively. The sensitivity of this approach was comparable to that of qPCR analysis. Chen et al. [146] developed a rapid detection method for SARS-CoV-2 that combines RPA with DNA–protein crosslinking chemiluminescence (DPCL), termed RPADPCL. In this method, the modified product was captured using streptavidin (SA)-labeled magnetic beads, followed by analysis with a chemiluminescence detector and a smartphone after the addition of a fluorescent substrate. This setup achieved a detection limit as low as six copies, highlighting its potential for rapid and sensitive diagnosis.

### 4.7. RPA Combined with Surface-Enhanced Raman Scattering (SERS) Detection

**Figure 6:**
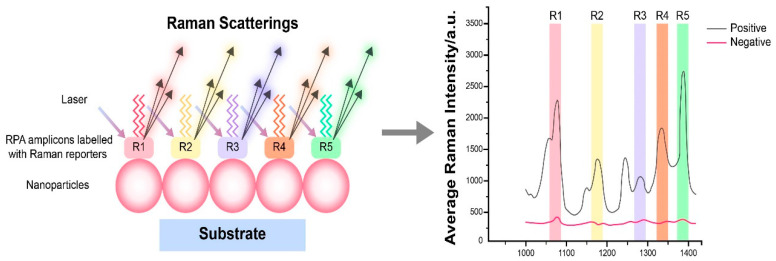
Schematic diagram of RPA combined with SERS detection.

SERS relies on two primary mechanisms: electromagnetic enhancement, which involves the local electric field enhancement on metal surfaces, and chemical enhancement, referring to electron transfer between metals and molecules [147]. SERS has emerged as a widely utilized technique for inelastic light scattering sensing. When molecules are adsorbed onto roughened metal surfaces, such as silver or gold nanoparticles (AuNPs), SERS signals can be amplified by factors of 10^8^ or more, facilitating the detection of even single molecules [148]. The detection principle is shown in Figure 6. Zhuang et al. [149] designed a microfluidic paper-based analytical device that integrates RPA with SERS and CRISPR/Cas12a, achieving a detection limit of approximately 3 to 4 CFU/mL for *Salmonella typhimurium* (*S. typhi*). Lau et al. [150] applied RPA-SERS to detect plant pathogens, successfully identifying as few as two copies of B. cinerea DNA. Additionally, Liu et al. [151] developed a novel SERS-based lateral flow (LF) strip biosensor combined with RPA, enabling the simultaneous detection of *Salmonella enterica* serotype *Enteritidis* and *Listeria monocytogenes*, with detection limits of 27 and 19 CFU/mL, respectively. Koo et al. [152] utilized isothermal reverse transcriptase polymeric amplification (RT-RPA) to amplify TMPRSS2-ERG transcripts, which are recurrent biomarkers for prostate cancer (PCa), and combined this with SERS for direct detection of the amplicons. This methodology successfully detected 10^3^ copies of TMPRSS2-ERG transcripts and has been effectively applied to clinical PCa urinary samples.

### 4.8. RPA Combined with Surface-Enhanced Infrared Absorption Spectroscopy (SEIRA)

SEIRA is an infrared spectroscopy technique that enhances the infrared signal of target molecules through molecular vibration coupled with surface equivalent excitation resonance. This enhancement can increase the infrared signal by factors ranging from 10^3^ to 10^6^ [153]. Gold and silver nanomaterials are commonly employed in SEIRA due to their high stability and favorable dielectric properties [154,155,156,157]. Yao et al. [23] utilized the SEIRA effect of gold nanoparticles to develop an RPA-based infrared spectral biosensor capable of detecting as few as 2.98 copies/μL of SARS-CoV-2 within 30 min. However, a notable limitation of this method is the requirement for the purification of RPA products, which introduces additional steps in the detection process [7].

### 4.9. RPA Combined with Microfluidic Technology

Due to its rapid reaction time, high sensitivity, and ability to operate under mild and constant temperature conditions, RPA is particularly well-suited for integration with microfluidic chips to develop point-of-care testing (POCT) technologies for pathogenic microorganisms [24]. The microfluidic chip integrates a microfluidic network composed of a micro-valve, micro-pump, micro-reactor, micro-channel, and other functional units, which can automatically perform multiple reaction steps such as the pretreatment, enrichment, reaction, labeling, and detection of test samples. This setup facilitates the closed, automated testing process of “sample input—result output” and significantly enhances testing efficiency. It also supports high-throughput, parallel and even multipath detection of multiple targets, greatly improving the overall detection capability [158]. Liu et al. [159] established a microfluidic-integrated lateral flow recombinase polymerase amplification (MI-LF-RPA) method that executes isothermal reverse transcription, amplification, labeling, and antibody binding of target genes directly on the chip. This method successfully demonstrated rapid and sensitive detection of SARS-CoV-2, achieving a detection limit of 1 copy/μL. The performance of the chip was further validated using clinically diagnosed COVID-19 cases, showing a sensitivity of 97% and a specificity of 100%. Additionally, a RPA chip developed by Tae Seok Seo’s research team at the Korea Institute of Science and Technology facilitates the simultaneous detection of multiple pathogens from a single sample, successfully identifying *Salmonella enterica*, Escherichia coli, and Vibrio parahaemolyticus [113]. Chen et al. [160] created a dish RPA chip specifically designed for the detection of common pathogenic microorganisms in urine. This chip can analyze two samples simultaneously, and each sample can screen for nine targets. It effectively detected Escherichia coli, Proteus mirabilis, Pseudomonas aeruginosa, and Staphylococcus aureus within 40 min, with a detection limit of 10^2^ CFU/mL. The detection limit for *Salmonella typhimurium* was found to be 10^3^ CFU/mL. Kim et al. [161] integrated lateral flow strips with the dish chip, enabling direct interpretation of results by the naked eye. The detection limits for *Salmonella* in phosphate-buffered saline (PBS) and dairy products were 10^1^ CFU/mL and 10^2^ CFU/mL, respectively, with detection achieved in just 30 min.

## 5. Conclusions and Future Prospects

RPA technology addresses several limitations of fluorescence quantitative PCR (qPCR), including the need for complex instrumentation and high costs. As a novel nucleic acid amplification technique, RPA offers significant advantages such as simple equipment, rapid reaction times, and high sensitivity. Currently, TwistDx™ provides a variety of RPA reaction kits designed for the detection of specific foodborne pathogens, including *Listeria monocytogenes*, *Campylobacter*, and *Salmonella enterica*. The company offers RPA reagents not only in liquid format but also in lyophilised pellet format, allowing field application. In addition, tailor-made devices and accessories for RPA reactions were developed. These devices and accessories enable incubation, dispensing, mixing, detection, power, and portability [44]. Research conducted both domestically and internationally indicates that RPA technology is poised for substantial advancement in POCT and in conjunction with other technologies over the coming years. A key feature of POCT is its simplified preprocessing methods, which enable the rapid acquisition of diagnostic results. To enhance its clinical application, the development of multiple detection platforms capable of simultaneously identifying various pathogens or biomolecules will be essential. With ongoing advancements in microfluidics and portable analytical devices, RPA is expected to see broader applicability, especially in field settings or resource-limited environments, facilitating expedited detection. In conclusion, RPA represents a promising technology with significant practical value following the advent of PCR. It is anticipated that through continuous exploration and innovation in RPA technology, diagnostic products based on isothermal nucleic acid amplification will proliferate. RPA will carve out its unique niche in medical diagnostics, leading to more frequent use in routine testing and making valuable contributions to the healthcare industry.

## Figures and Tables

**Table 1:** The application of RPA technology in the field of pathogen detection.

Pathogenic Agent	Detection Method	LOD	Ref
Porcine circovirus 2	qRPA	100 copies	[58]
Porcine parvovirus	qRPA RPA-LFD	300 copies 400 copies	[59]
H5N1 avian influenza	RT-qRPA	1 copies	[60]
White spot syndrome virus	qRPA	10 copies	[61]
H7N9 avian influenza	qRPA	10~100 copies	[62]
Dengue virus	qRPA	10 copies	[63]
Yellow fever virus	LF-RPA	21 copies	[64]
Plum pox virus	LF-RPA	1 fg	[65]
Little cherry virus 2	LF-RPA	100 fg	[66]
Yam mosaic virus	qRPA	14 pg	[67]
Infectious hypodermal and hematopoietic necrosis	qRPA	4 copies	[68]
